# Decreased Cerebral Blood Flow and Delayed Arterial Transit Are Independently Associated With White Matter Hyperintensity

**DOI:** 10.3389/fnagi.2022.762745

**Published:** 2022-05-27

**Authors:** Ruiting Zhang, Peiyu Huang, Shuyue Wang, Yeerfan Jiaerken, Hui Hong, Yao Zhang, Xinfeng Yu, Min Lou, Minming Zhang

**Affiliations:** ^1^Department of Radiology, School of Medicine, The Second Affiliated Hospital of Zhejiang University, Hangzhou, China; ^2^Department of Neurology, School of Medicine, The Second Affiliated Hospital of Zhejiang University, Hangzhou, China

**Keywords:** cerebral blood flow, arterial transit, white matter hyperintensities, lacunes, cerebral small vessel disease

## Abstract

**Aim:**

White matter hyperintensities (WMH) and lacunes were important features of cerebral small vessel disease (CSVD), which contributes to 25% of ischemic strokes and 45% of dementias. Currently, the underlying mechanisms of WMH and lacunes are not clear, and the role of hemodynamic changes is not fully investigated. In this study, we aimed to measure the cerebral blood flow (CBF) and arterial transit in CSVD patients and to investigate their association with WMH and lacunes.

**Methods:**

We retrospectively analyzed the prospectively collected database of CSVD patients. Ninety-two CSVD patients with complete imaging data were included. We used arterial spin labeling (ASL) with post-labeling delay time (PLD) of 1,525 ms and 2,025 ms to measure CBF respectively, and the difference between CBF_PLD1.5_ and CBF_PLD2.0_ was recorded as δCBF. We performed regression analysis to understand the contribution of CBF, δCBF to CSVD imaging markers.

**Results:**

We found that CBF derived from both PLDs was associated with WMH volume and the presence of lacune. CBF_PLD1.5_ was significantly lower than CBF_PLD2.0_ in CSVD patients, and δCBF was correlated with WMH volume but not the presence of lacune. Furthermore, CBF_PLD2.0_ and δCBF were both associated with WMH in multiple regression analyses, suggesting an independent effect of delayed arterial transit. On an exploratory basis, we also investigated the relationship between venous disruption on δCBF, and we found that δCBF correlated with deep medullary veins score.

**Conclusion:**

Both CBF and arterial transit were associated with WMH. ASL with multiple PLDs could provide additional hemodynamic information to CSVD-related studies.

## Introduction

White matter hyperintensities (WMHs) and lacunes of presumed vascular origin are neuroimaging features of the cerebral small-vessel disease (CSVD) and are closely related to future stroke and dementia (Debette et al., 2019). Currently, their causes have not been completed understood. Possible mechanisms including hypoperfusion, impaired cerebral vascular reactivity, and the disruption of the blood-brain barrier had been brought out during the past decades (Wardlaw et al., 2019). Among them, chronic hypoperfusion of the white matter had been investigated by many groups (Shi et al., 2016), and it is considered as one of the main reasons for the occurrence of WMH and lacunes.

Many previous studies investigated the relationship between CBF and CSVD using different imaging methods (Shi et al., 2016). Among them, arterial spin labeling (ASL) has been highly recommended since it is noninvasive and can quantify regional cerebral blood flow (CBF) values with good reproducibility (Haller et al., 2016). Importantly, appropriate choice of post-labeling delay time (PLD) is essential for CBF measurement because it is possible that the labeled bolus may not have reached or have passed the imaging plane, both leading to underestimation of CBF (Lou et al., 2019). CSVD patients might need longer PLD than normal elderly people as small vessel injury might be associated with delayed arterial transit (Huynh et al., 2008). Nevertheless, using a single long PLD (2,000 ms or 2,500 ms) is not ideal for studying patients in a large cohort with different degrees of vascular impairment, as CBF might be underestimated in relatively healthy subjects (Hu et al., 2019). Currently, there is no recommended PLD for CSVD patients.

Additionally, recent studies suggest that intracranial hemodynamic changes may contribute to CSVD (Blair et al., 2020). When total cerebral blood flow remains the same, vascular resistance may change significantly. Resistance might come from arteriosclerosis and lipohyalinosis of the arterioles (Humphreys et al., 2021), as well as the collagenosis of the venules (Keith et al., 2017), since both of them could lead to stenosis. With perfusion imaging at multiple PLDs, one could observe the trend from CBF values at different delays or estimate arterial arrival time to quantify the delay of blood flow, although currently there are also some challenges in accurately fitting the kinetic model from multi-delay ASL.

Until now, few studies have investigated CBF changes in CSVD patients using multiple PLDs, and the association between delayed arterial transit and CSVD is yet to be verified. According to the consensus of the ISMRM Perfusion Study Group and the European Consortium for ASL in Dementia, a 1,800 ms PLD was recommended for healthy adults under 70 years old and a 2,000 ms PLD was recommended for adult clinical patients (Alsop et al., 2015). Therefore, in the current study, we first chose a 2,025 ms PLD in order to follow the general recommendation, and then we chose a 1,525 ms PLD in order to highlight the effect of arterial transit time. We aimed to: (1) investigate brain perfusion derived from ASL with two PLDs (1,525 ms/2,025 ms) in CSVD patients; (2) evaluate the relationship between CBF, δCBF (the subtraction of CBF_PLD2.0_ and CBF_PLD1.5_), and CSVD imaging markers; and (3) explore the influence of venous disruption on δCBF. We hypothesized that CBF derived from the two PLDs would be different, and both CBFs and δCBF would be associated with WMH and lacunes.

## Materials and Methods

### Study Subjects

We retrospectively reviewed the data of our prospective collected CSVD database (CSVD defined as the presence of lacunes and/or WMH on MRI) admitted to the neurology department of our hospital from November 2017 to July 2020. The main reasons for referral to the hospital were acute or subacute symptoms of CSVD, and the diagnosis of patients included lacunar stroke, transient ischemic attack, vascular dementia, etc. The imaging data of patients with acute lacunar infarct were acquired at least 6 months after onset. The inclusion criteria were: (1) age above 40; (2) with complete multi-modality MRI data (including ASL with two PLDs); and (3) had written informed consent. Exclusion criteria were: (1) patients with secondary causes of white matter lesions, such as demyelinating, infectious, metabolic, and immunological diseases; (2) patients with abnormal brain MRI findings such as head trauma, hemorrhage, infarction (except lacunes), and other space-occupying lesions; (3) patients with definitive peripheral neuropathy, spinal cord disease; (4) poor image quality that affected further analysis. We retrieved the demographic, clinical, laboratory, and imaging data of all patients; and (5) patients who showed significant intracranial large artery stenosis (>50%) on MRA.

### MRI Protocol

All subjects underwent multi-model MRI by a 3.0T MR (Discovery MR750, GE Healthcare, USA) scanner using an 8-channel brain phased array coil. The scanning sequences included 3D sagittal T1 (3D-T1) weighted imaging, T2 fluid attenuated inversion recovery (FLAIR) imaging, 3D arterial spin labeling (ASL) imaging with two PLDs, time-of flight magnetic resonance angiography (TOF-MRA) and susceptibility-weighted imaging (SWI). In order to minimize head motion, foam pads were inserted into the space between the subject’s head and the MRI head coil. The pulsed-continuous ASL data were acquired using a 3D spiral readout fast spin-echo sequence with background suppression. The parameters were: TR/TE = 4,611/10.5 ms, PLD = 1,525 ms or 2,025 ms, Label duration = 1,500 ms, flip angle = 111°, slice thickness = 4 mm, matrix = 128 × 128, FOV = 24 cm. The scan time is 4 min 28 s for the PLD 1,525 ms, and 4 min 40 s for the PLD 2,025 ms. 3D-T1 was acquired using spoiled gradient echo sequence with repetition time = 7.3 ms, echo time = 3.0 ms, inversion time = 450 ms, flip angle = 8°, slice thickness = 1 mm, matrix = 250 × 250, FOV = 25 cm × 25 cm. T2 FLAIR acquisition parameters were: repetition time = 8,400 ms, echo time = 150 ms, flip angle = 90°, FOV = 24 cm × 24 cm, matrix size = 256 × 256, inversion time = 2,100 ms, slice thickness = 4.0 mm with no gap between slices. TOF-MRA consisted of three slabs with TR = 21 ms, TE = 2.4 ms, flip angle = 20°, and slice thickness = 1.4 mm. The SWI sequence was acquired in an axial orientation parallel to the anterior commissure to posterior commissure line, using a three-dimension multi-echo gradient-echo sequence with eight equally spaced echoes: first echo time = 4.5 ms, echo spacing = 3.65 ms, last echo time = 30 ms, repetition time = 34 ms, FOV = 24 cm × 24 cm, matrix size = 416 × 384, flip angle = 20°, slice thickness = 2.0 mm with no gap between slices, and the in-plane spatial resolution interpolated into 0.4688 mm/pixel × 0.4688 mm/pixel. The magnitude and phase images were reconstructed and saved.

### Assessments of WMH

The axial T2 FLAIR images were processed for the quantification of WMH volumes in a semi-automatic way. Specifically, WMH segmentation was performed using the lesion prediction algorithm (LPA) within the Lesion Segmentation Toolbox in SPM12. A threshold of 0.5 was applied to the probabilistic lesion map to derive a binarized WMH image. Then the segmented lesions were visually checked and manually corrected by two experienced neuro-radiologists (RZ and PH, both with 6 years of neuroimaging review experience) who were blinded to all other imaging and clinical data. The manual correction process included: (1) correction of non-white matter areas labeled as WMH; and (2) WMH area falsely labeled as WMH or normal appearing white matter (NAWM) falsely labeled as WMH. WMH and NAWM masks were obtained for further analyses. After manual corrections, the volume of WMH was extracted for each subject.

### ASL and CBF Analysis

After the acquisition, the scanner would output an averaged subtraction image (tag-control) and a proton density image for each subject. The images were transformed into NIFTI format and processed using Bayesian Inference for Arterial Spin Labeling MRI (BASIL^1^). The processing steps included: (1) anatomical processing of T1 structural images using fsl_anat, which is a complex pipeline involving image reorientation, bias-field correction, registration to standard space, brain extract, tissue-type segmentation, etc; (2) registration of ASL images to structural images using boundary-based registration (BBR) cost function; (3) calibration to produce images of absolute perfusion (in ml/100 g/min), using the proton density image and methods in a consensus article (Alsop et al., 2015); and (4) partial volume correction to avoid contamination among different tissue types.

After these steps, calibrated CBF maps were produced in three spaces: native ASL space, structural space, and standard space. Images in the structural space were used for further analysis. We visually assessed the results to ensure that the T1 and CBF images were well-aligned and reasonable CBF maps were generated. CBF maps derived from PLD 1,525 (CBF_PLD1.5_) were subtracted from CBF maps derived from PLD 2,025 (CBF_PLD2.0_) to generate δCBF maps.WMH and NAWM masks were co-registered to T1 images and used for extracting regional CBF values.

### Measurement of Deep Medullary Veins (DMVs)

The raw data were transferred to a separate workstation (ADW4.4, GE), and a custom-built program was used to reconstruct the magnitude and phase images. We assessed DMVs on SWI phase images from the level of the lateral ventricles first appeared (top level) to the level of caudate first appeared (bottom level), which were about five consecutive periventricular slices (10 mm thick), considering these slices cover a large portion of DMVs. According to medullary venous anatomy, six subregions including the frontal region, parietal region and occipital region (bilateral, respectively) were separated on the above five slices and the characteristics of the DMVs were then evaluated in each subregion, respectively.

As described in our previous study (Zhang et al., 2017), we used the four-point scoring method to evaluate DWVs: 0 score—each vein was continuous and had a homogeneous signal; 1 score—each vein was continuous, but one or more than one vein had inhomogeneous signal; 2 score—one or more than one vein was not continuous; and 3 score—No observed vein was found continuous. The total DWVs score is the sum of the six subregions’ scores. Two neuroradiologists (RZ and PH), who were completely blinded to the subjects’ clinical data and disease state, assessed the vascular changes. The interobserver intraclass correlation coefficient (ICC) was 0.89 for grading of DMVs. ICC was described in detail elsewhere.

### Evaluation of Microbleeds

SWI magnitude images were used to identify microbleeds. Briefly, microbleeds should be small, rounded, or circular, well-defined hypointense lesions within brain parenchyma with clear margins ranging from 2 to 10 mm in size. Signal voids caused by sulcal vessels, calcifications, choroid plexus, and low-signal averaging from adjacent bone were excluded.

### Evaluation of Lacunes of Presumed Vascular Origin (Simplified as Lacunes)

T2 FLAIR images were used to identify lacunes. A lacune was defined as a round or ovoid, subcortical, fluid-filled (similar signal as CSF) cavity on MRI and with a diameter of 3–15 mm, which was different from the enlarged Virchow–Robin spaces by the size, shape, and rim.

### Statistical Analysis

All metric and normally distributed variables were reported as mean ± standard deviation, and non-normally distributed variables were reported as median (25th–75th percentile). Categorical variables were presented as frequency (percentage). First, we performed paired Student’s *t*-tests between CBF derived from different PLDs and analyzed the association between CBF and δCBF. Second, we performed linear regression and logistic regression analysis to understand the contribution of CBF and δCBF to CSVD imaging markers. Each CSVD marker was set as dependent variable and each of the CBF indices was set as independent variable, controlling for age and sex (Model 1). Third, we added hypertension, diabetes, hyperlipidemia, and smoking to the first model (Model 2) to exclude the influence of these risk factors. Finally, to disentangle the independent contribution of decreased CBF and delayed arterial transit, we put CBF_PLD1.5_ or CBF_PLD2.0_ together with δCBF and other variables (age, sex, hypertension, diabetes, hyperlipidemia, and smoking) to predict CSVD markers (Model 3). Since δCBF could reflect arterial transit delay, which might be affected by venous outflow, we further tested whether δCBF was associated with DMVs disruption. All analyses were performed blinded to participants’ identifying information. A *p*-value of <0.05 was considered to be statistically significant. All statistical analysis was performed with SPSS package (22.0 for Windows).

## Results

### Subjects’ Characteristics

One-hundred and three patients participated in our study. Among them, three patients had poor image quality, one patient developed a large infarct and seven patients had significant intracranial large artery stenosis. Finally, 92 patients were included in the present study.

Among the 92 patients, two patients had TIA, 59 patients had a lacunar stroke, six patients had dementia, four patients had vertigo, six patients had dizziness, two patients had headache, and 13 patients were found to have white WMH/lacune during imaging examination for other diseases.

Characteristics of the study samples are presented in Table 1. Briefly, patients’ median normalized WMH volume was 0.9% (0.3%–2.0%). Forty-eight (52.2%) subjects presented with microbleeds, and 44 (47.8%) subjects presented with lacunas.

**Table 1:** Subject’s characteristics (revised).

**Variables**	***n* = 92**
Age (Y)	65.2 ± 9.5
Female, *n* (%)	41 (45%)
Vascular risk factors, *n* (%)	
Hypertension	61 (66%)
Diabetes mellitus	14 (15%)
Hyperlipidemia	12 (13%)
Smoking	19 (21%)
Radiology data	
Normalized WMH volume, %	0.9 (0.3–2.0)
Number of microbleeds	1 (0–7)
Number of lacunes	1 (0–3)

*WMH, white matter hyperintensities*.

### Different CBF Values Were Derived From Different PLDs in CSVD Patients

Compared with mean CBF_PLD1.5_, mean CBF_PLD2.0_ was significantly higher in all brain regions, including gray matter, NAWM and WMH (Table 2). δCBF was associated with CBF_PLD1.5_, but such association did not exist for CBF_PLD2.0_ (Supplementary Table S1).

**Table 2:** Different CBF values were derived from different PLDs in CSVD patients (revised).

**CBF (ml/100 g/min)**	**PLD = 1,525 ms**	**PLD = 2,025 ms**	***p* value**
Gray matter	35.37 ± 7.66	38.04 ± 6.69	< 0.001
NAWM	26.39 ± 4.95	28.16 ± 4.37	< 0.001
WMH	19.26 ± 4.67	20.69 ± 3.91	< 0.001

*CBF, cerebral blood flow; NAWM, normal appearing white matter; WMH, white matter hyperintensities; PLD, post-labeling delay time*.

### CBF Correlated With CSVD Imaging Markers

As shown in Table 3; CBF_PLD1.5_ and CBF_PLD2.0_ were both correlated with normalized WMH volumes after adjusting for age and sex (model 1 in Supplementary Table S2), except for CBF_PLD2.0_ in NAWM. These associations remained significant after adding hypertension, diabetes, hyperlipidemia, and smoking into the regression model (model 2 in Table 3). Similarly, except for CBFs in NAWM, CBF_PLD1.5_ and CBF_PLD2.0_ were correlated with the presence of lacune after adjusting for age and sex (model 1), as well as further adjusting for hypertension, diabetes, hyperlipidemia, and smoking (model 2).

**Table 3:** Regression analyses between CBF and CSVD imaging markers (revised).

	**Lg (normalized WMH volume)**		**The presence lacune**	
	**Standardized β**	***P***	**OR**	***P***
CBF_PLD1.5_ in gray matter	−0.410	<0.001	0.929	0.036
CBF_PLD1.5_ in NAWM	−0.253	<0.017	0.911	0.074
CBF_PLD1.5_ in WMH	−0.483	<0.001	0.880	0.024
CBF_PLD2.0_ in gray matter	−0.307	<0.004	0.926	0.049
CBF_PLD2.0_ in NAWM	−0.098	<0.360	0.943	0.299
CBF_PLD2.0_ in WMH	−0.409	<0.001	0.877	0.045

*CBF, cerebral blood flow; NAWM, normal appearing white matter; WMH, white matter hyperintensities; PLD, post-labeling delay time. Normalized WMH volume and the presence of lacune were set as dependent variables and each of the CBF indices was set as independent variables, controlling for age, sex, hypertension, diabetes, hyperlipidemia, and smoking*.

### δCBF Correlated With WMH Volumes but Not the Presence of Lacune

**Figure 1:**
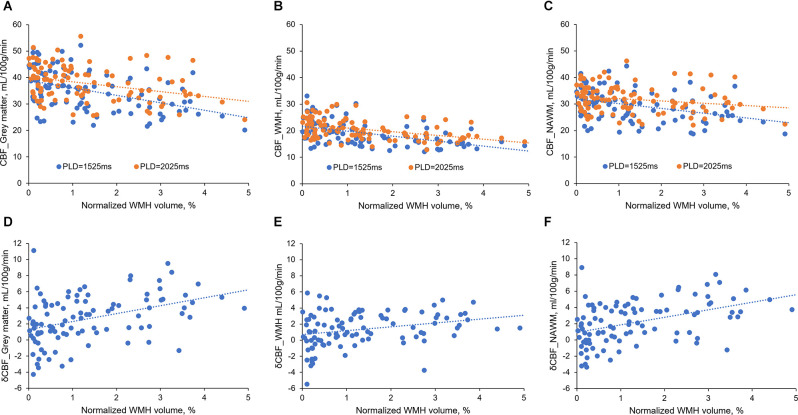
The relationship between CBFs and normalized WMH volume. **(A–C)** CBFs correlated with normalized WMH volume. The difference between the two CBF values became larger when WMH volumes increased. **(D–F)** δCBF in all three brain regions was associated with normalized WMH volume.

Interestingly, during analyzing the relationship between CBF and normalized WMH volumes, we found that the difference between the two CBF values became larger when WMH volume increased (Figures 1A–C). Therefore, we further investigated the relationship between δCBF and WMH volumes, and we found that δCBF in all three brain regions was associated with normalized WMH volume (Figures 1D–F). After adjusting for age, sex (model 1 in Supplementary Table S3), hypertension, diabetes, hyperlipidemia, and smoking (model 2 in Table 4), the association remained significant.

**Table 4:** Linear regression analyses between δCBF and CSVD imaging markers (revised).

	**Lg (normalized WMH volume)**		**The presence lacune**	
	**Standardized β**	***P***	**OR**	***P***
δCBF in gray matter	0.331	<0.002	1.708	0.346
δCBF in NAWM	0.372	<0.001	1.236	0.059
δCBF in WMH	0.307	<0.004	1.162	0.200

*δCBF, the difference between CBF value derived from two PLDs (CBF_PLD2.0_-CBF_PLD1.5_); NAWM, normal appearing white matter; WMH, white matter hyperintensities. Normalized WMH volume and the presence of lacune were set as dependent variables and δCBF was set as an independent variable, controlling for age, sex, hypertension, diabetes, hyperlipidemia and smoking*.

We also tested the relationship between δCBF and the presence of lacune. We found that only δCBF in NAWM was associated with the presence of lacune, and the association became non-significant after adjusting for hypertension, diabetes, hyperlipidemia, and smoking (Table 4).

### δCBF and CBF_PLD2.0_ Were Independently Associated With CSVD Markers

When putting δCBF and CBF together into the regression model, δCBF remained significant with CBF_PLD2.0_ in all brain regions (Table 5), but with CBF_PLD1.5_ the δCBF was only significant in NAWM. Similarly, δCBF was associated with WMH but not lacunes.

**Table 5:** Linear regression analyses between CBF, δCBF, and CSVD imaging markers (revised).

**Model 3**	**Lg (WMH volume)**		**The presence lacune**	
	**Standardized β**	***P***	**OR**	***P***
CBF_PLD1.5_ in gray matter	−0.322	<0.006	0.928	0.056
δCBF in gray matter	0.181	<0.107	0.990	0.916
CBF_PLD1.5_ in NAWM	−0.106	<0.348	0.940	0.282
δCBF in NAWM	0.322	<0.006	1.167	0.213
CBF_PLD1.5_ in WMH	−0.452	<0.001	0.882	0.056
δCBF in WMH	0.058	<0.607	1.009	0.950
CBF_PLD2.0_ in gray matter	−0.281	<0.006	0.928	0.056
δCBF in gray matter	0.308	<0.002	1.068	0.427
CBF_PLD2.0_ in NAWM	−0.094	<0.348	0.940	0.282
δCBF in NAWM	0.371	<0.001	1.242	0.057
CBF_PLD2.0_ in WMH	−0.379	<0.001	0.882	0.056
δCBF in WMH	0.262	<0.007	1.144	0.266

*δCBF, the difference between CBF value derived from two PLDs (CBF_PLD2.0_-CBF_PLD1.5_); NAWM, normal appearing white matter; WMH, white matter hyperintensities. Normalized WMH volume and the presence of lacune were set as dependent variables and δCBF was set as an independent variable, controlling for age, sex, hypertension, diabetes, hyperlipidemia, smoking, and CBF _PLD1.5_/CBF _PLD2.0_ (Model 3)*.

### The Relationship Between DMVs Score and δCBF

The results showed that δCBF in gray matter was correlated with the total DMVs score (Standardized *β* = 0.252, *P* = 0.016), and δCBF in NAWM tended to be correlated with total DMVs score (Standardized *β* = 0.194, *P* = 0.067), while the correlation between δCBF in WMH and DMVs score was non-significant (Standardized *β* = 0.154, *P* = 0.146).

## Discussion

In the current study, we found that CBFs derived from both PLDs were associated with WMH volume and the presence of lacune. CBF_PLD1.5_ was significantly lower than CBF_PLD2.0_ in CSVD patients, and δCBF was correlated with WMH volume but not the presence of lacune. Furthermore, CBF_PLD2.0_ and δCBF were both associated with WMH in multiple regression analyses, suggesting an independent effect of delayed arterial transit. On an exploratory basis, we found that δCBF correlated with venous disruption.

We found that lower CBF was associated with higher WMH volume and more lacunes, and these relationships were independent of vascular risk factors. These results were consistent with most previous studies, especially in studies that included CSVD patients or elderly people with moderate-severe WMH lesion load (Promjunyakul et al., 2015; Shi et al., 2016; Zhou et al., 2020). In studies that enrolled community-dwelling elderly individuals, the relationship between CBF and WMH tends to be weaker. For example, one previous study which included community-dwelling elderly individuals with hypertension (median WMH volume = 6.5 ml) found that WMH severity was only associated with CBF in the WMH region but not NAWM or gray matter (van Dalen et al., 2016). One possible reason could be that CBF in NAWM and gray matter might retain since the integrity of the small vessels in these regions was relatively intact in the early phase of CSVD (Promjunyakul et al., 2016). Another possible reason is that the mechanisms of WMH might be distinct in different populations. Although ischemia is a major pathology of WMH, the occurrence of WMH in community cohort might also be ascribed to other non-ischemic reasons, such as the retention of interstitial fluid caused by venous disruption or PVS obstruction (Huang et al., 2021; Zhang et al., 2021).

**Figure 2:**
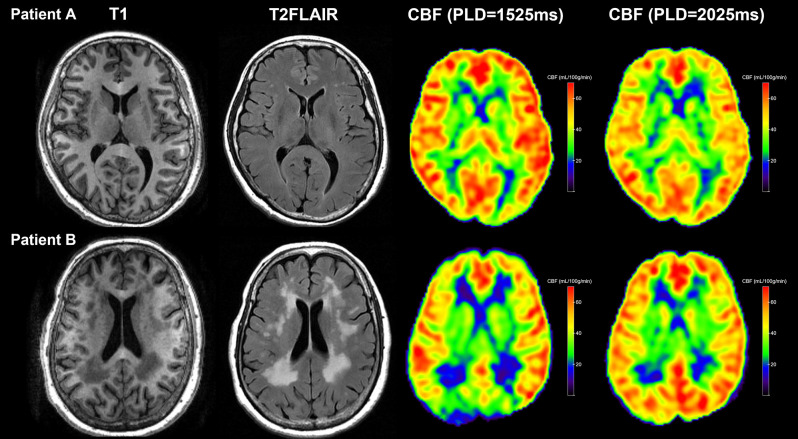
Two representative patients with different WMH burdens. Upper row: Patient A had a low WMH burden and presented with higher global CBF_PLD1.5_ than CBF_PLD2.0_. Lower row: Patient B had a high WMH burden and presented with lower global CBF_PLD1.5_ than CBF_PLD2.0_.

Due to the arterial arrival delay, here CBF_PLD1.5_ was significantly lower than CBF_PLD2.0_ in most subjects. We can also see that CBF_PLD1.5_ but not CBF_PLD2.0_ was associated with δCBF, confirming the influence of PLD on the measurement of CBF. Although this would be expected, because the real arterial arrival time still depended on each subject’s age and clinical status, the δCBF could reflect such differences. Indeed, some of our subjects had relatively young age and less vascular burden, so they had higher CBF when the PLD was short (Figure 2, upper row). On the contrary, those with large areas of WMH had significantly higher CBF when the PLD was long (Figure 2, lower row). Therefore, we believe larger δCBF was associated with longer arterial transit time.

Notably, δCBF was associated with WMH volume (Figure 1), indicating the contribution of delayed arterial transit. Postmortem studies have found small vessel wall thickening and stenosis in WMH areas, which might lead to increased vascular resistance and reduced blood flow velocity (Gouw et al., 2011). Further, the reduced blood velocity might cause platelet aggregation, local accumulation of inflammatory mediators, leukocyte activation, and endothelial dysfunction (Diamond, 2016; Jensen et al., 2017), resulting in the development of WMH. Importantly, when we put δCBF and CBF together into the regression model, δCBF and CBF_PLD2.0_ were independently associated with CSVD markers. This result confirmed the above-mentioned mechanisms, suggesting that in addition to ischemia, the reduced blood velocity may indeed have independent contributions. However, we had not found a significant association between lacunes and δCBF. Considering that lacune represents an end-stage of regional hypoperfusion when the small vessels finally occluded and blood flow is not able to arrive even with a longer PLD, the lack of association between lacunes and δCBF could be understood.

Additionally, we discovered that δCBF is related to venous disruption in CSVD patients. Venous collagenosis is an important feature of CSVD, which could lead to intramural thickening, stenosis, and ultimately luminal occlusion, resulting in the elevation of venous pressure, thereby associated with the reduction of blood flow velocity. Experimental studies in vein occlusion models demonstrated that the growth of the venous thrombi coincided with a decrease in regional CBF (Nakase et al., 1997), and clinical studies also demonstrated that central retinal vein occlusion could cause the delay of arterial transit (Crama et al., 2010).

Our study had limitations. First, as our cohort only included Chinese patients at a single institution and it may not represent the full spectrum of CSVD patients, the generalizability of our results requires confirmation and extension in larger and multicenter cohorts. Second, the patients in our cohort were recruited based on imaging evaluation, and heterogeneity existed between patients with stroke/TIA, cognitive impairments, and other clinical manifestations. Future studies are needed to detangle the differences between CSVD patients with different symptoms. Third, we didn’t analyze small artery stenosis. With the help of advanced imaging methods like high-resolution vessel wall imaging, it would be possible to find out the direct relationship between blood flow velocity and small artery stenosis.

In summary, we found the effect of δCBFon WMH and demonstrated the presence of delayed arterial transit in CSVD patients. Besides, our results showed that CBF_PLD1.5_ was related to δCBF, and also more related to WMH and lacune than CBF _PLD2.0_, indicating that CBF derived from a shorter PLD might highlight the hemodynamic changes and be more clinically important. For a group of patients with heterogeneity, using a single PLD might not be ideal to capture all the information, and multiple PLDs could help to detangle the complex hemodynamic changes.

## Data Availability Statement

The raw data supporting the conclusions of this article will be made available by the authors, upon reasonable request to the corresponding author Minming Zhang, zhangminming@zju.edu.cn.

## Ethics Statement

The studies involving human participants were reviewed and approved by the medical ethics committee of the Second Affiliated Hospital, Zhejiang University School of Medicine. The patients/participants provided their written informed consent to participate in this study. All clinical investigation has been conducted according to the principles expressed in the Declaration of Helsinki.

## Author Contributions

RZ, PH, and MZ were responsible for the study concept and design. YJ, SW, HH, YZ, XY, and ML contributed to the acquisition of imaging data. RZ and PH performed data analysis and interpreted the findings. RZ and PH drafted the manuscript. MZ provided critical revision of the manuscript for important intellectual content. All authors contributed to the article and approved the submitted version.

## Conflict of Interest

The authors declare that the research was conducted in the absence of any commercial or financial relationships that could be construed as a potential conflict of interest.

## Publisher’s Note

All claims expressed in this article are solely those of the authors and do not necessarily represent those of their affiliated organizations, or those of the publisher, the editors and the reviewers. Any product that may be evaluated in this article, or claim that may be made by its manufacturer, is not guaranteed or endorsed by the publisher.

## Footnotes

^1^ https://fsl.fmrib.ox.ac.uk/fsl/fslwiki/BASIL
