# Supplementary material for: Decreased Cerebral Blood Flow and Delayed Arterial Transit Are Independently Associated With White Matter Hyperintensity
Source: Front Aging Neurosci. 2022 May 27;14:762745. doi: 10.3389/fnagi.2022.762745 (PMC9197206; doi:10.3389/fnagi.2022.762745)
Supplement: Supplementary file 1 [file Data_Sheet_1.docx]

**Supplementary Materials**

**Table S1 The association between CBF_PLD1.5_, CBF_PLD2.0_ and δCBF**

|  | **CBF_PLD1.5_ (Standardized β, P)** | **CBF_PLD2.0_ (Standardized β, P)** |
| --- | --- | --- |
| **Grey matter** | -0.498 (<0.001) | -0.119 (0.260) |
| **NAWM** | -0.467 (<0.001) | -0.012 (0.907) |
| **WMH** | -0.554 (<0.001) | -0.122 (0.246) |

CBF: cerebral blood flow; NAWM: normal appearing white matter; WMH: white matter hyperintensities; PLD: post labeling delay time.

**Table S2 Regression analyses between CBF and CSVD imaging markers (revised)**

|  | **Lg (normalized WMH volume)** | | **The presence of lacune** | |
| --- | --- | --- | --- | --- |
|  | Standardized β | P | OR | P |
| CBF_PLD1.5_ in grey matter | -0.422 | < 0.001 | 0.926 | 0.015 |
| CBF_PLD1.5_ in NAWM | -0.266 | 0.010 | 0.912 | 0.050 |
| CBF_PLD1.5_ in WMH | -0.500 | < 0.001 | 0.881 | 0.015 |
| CBF_PLD2.0_ in grey matter | -0.307 | 0.003 | 0.927 | 0.031 |
| CBF_PLD2.0_ in NAWM | -0.101 | 0.330 | 0.946 | 0.274 |
| CBF_PLD2.0_ in WMH | -0.410 | < 0.001 | 0.886 | 0.039 |

CBF: cerebral blood flow; NAWM: normal appearing white matter; WMH: white matter hyperintensities; PLD: post labeling delay time.

Normalized WMH volume and the presence of lacune were set as dependent variables and each of the CBF indices was set as independent variables, controlling for age and sex.

**Table S3 Linear regression analyses between δCBF and CSVD imaging markers (revised)**

|  | **Lg (normalized WMH volume)** | | **The presence of lacune** | |
| --- | --- | --- | --- | --- |
|  | Standardized β | P | OR | P |
| δCBF in grey matter | 0.361 | < 0.001 | 1.110 | 0.166 |
| δCBF in NAWM | 0.401 | < 0.001 | 1.272 | 0.025 |
| δCBF in WMH | 0.324 | 0.002 | 1.186 | 0.118 |

δCBF: the difference between CBF value derived from two PLDs (CBF_PLD2.0_-CBF_PLD1.5_); NAWM: normal appearing white matter; WMH: white matter hyperintensities.

Normalized WMH volume and the presence of lacune were set as dependent variables and δCBF was set as independent variable, controlling for age and sex.
